# Inhibition of CIN85-Mediated Invasion by a Novel SH3 Domain Binding Motif in the Lysyl Oxidase Propeptide

**DOI:** 10.1371/journal.pone.0077288

**Published:** 2013-10-22

**Authors:** Seiichi Sato, Yingshe Zhao, Misa Imai, Philip C. Simister, Stephan M. Feller, Philip C. Trackman, Kathrin H. Kirsch, Gail E. Sonenshein

**Affiliations:** 1 Department of Developmental, Molecular and Chemical Biology, Tufts University School of Medicine, Boston, Massachusetts, United States of America; 2 Department of Biochemistry, Boston University School of Medicine, Boston, Massachusetts, United States of America; 3 Department of Oncology, Weatherall Institute of Molecular Medicine, University of Oxford, Oxford, United Kingdom; 4 Section Tumor Biology, Institute of Molecular Medicine, Martin-Luther-University Halle-Wittenberg, Halle (Saale), Germany; 5 Division of Oral Biology, Boston University Henry M. Goldman School of Dental Medicine, Boston, Massachusetts, United States of America; Wayne State University School of Medicine, United States of America

## Abstract

The lysyl oxidase gene inhibits Ras signaling in transformed fibroblasts and breast cancer cells. Its activity was mapped to the 162 amino acid propeptide domain (LOX-PP) of the lysyl oxidase precursor protein. LOX-PP inhibited the Her-2/Ras signaling axis in breast cancer cells, and reduced the Her-2-driven breast tumor burden in a xenograft model. Since its mechanism of action is largely unknown, co-affinity-purification/mass spectrometry was performed and the “Cbl-interacting protein of 85-kDa” (CIN85) identified as an associating protein. CIN85 is an SH3-containing adapter protein that is overexpressed in invasive breast cancers. The CIN85 SH3 domains interact with c-Cbl, an E3 ubiquitin ligase, via an unconventional PxxxPR ligand sequence, with the highest affinity displayed by the SH3-B domain. Interaction with CIN85 recruits c-Cbl to the AMAP1 complex where its ubiquitination activity is necessary for cancer cells to develop an invasive phenotype and to degrade the matrix. Direct interaction of LOX-PP with CIN85 was confirmed using co-immunoprecipitation analysis of lysates from breast cancer cells and of purified expressed proteins. CIN85 interaction with c-Cbl was reduced by LOX-PP. Domain specific CIN85 regions and deletion mutants of LOX-PP were prepared and used to map the sites of interaction to the SH3-B domain of CIN85 and to an epitope encompassing amino acids 111 to 116 of LOX-PP. Specific LOX-PP point mutant proteins P111A and R116A failed to interact with CIN85 or to compete for CIN85 binding with c-Cbl. Structural modeling identified a new atypical PxpxxRh SH3-binding motif in this region of LOX-PP. The LOX-PP interaction with CIN85 was shown to reduce the invasive phenotype of breast cancer cells, including their ability to degrade the surrounding extracellular matrix and for Matrigel outgrowth. Thus, LOX-PP interacts with CIN85 via a novel SH3-binding motif and this association reduces CIN85-promoted invasion by breast cancer cells.

## Introduction

Lysyl oxidase (LOX) (protein-6-oxidase; EC 1.4.3.13) is a key extracellular enzyme that controls collagen and elastin crosslinking, which is required for the biosynthesis of functional extracellular matrices. The *LOX* gene was isolated as the *ras recision* gene (*rrg*) with the ability to inhibit the transforming activity of the H-*Ras* oncogene in fibroblasts [1]. Ectopic *LOX* gene expression in gastric cancer cells inhibits tumor formation in nude mice [2] and reduces *LOX* expression has been reported in many carcinomas (reviewed in [3]). Lysyl oxidase is synthesized and secreted as a 50-kDa inactive pro-enzyme, which is processed by proteolytic cleavage to a functional 32-kDa active enzyme (LOX) and an 18-kDa propeptide (LOX-PP). The Ras-inhibitory activity was mapped to the LOX-PP domain. LOX-PP inhibits Ras signaling and the transformed phenotype in Ras-transformed NIH 3T3 fibroblasts [4], and in Her-2/neu-driven NF639 breast cancer cells [5]. Ectopic LOX-PP expression in NF639 or MiaPaCa2 pancreatic cancer cells reduces tumor xenograft formation in nude mice [5]–[7], and prevented growth of pre-existing NF639 tumors [8]. The mechanisms by which LOX-PP exerts these anticancer effects are only beginning to be understood. Notably, LOX-PP attenuates fibronectin-mediated integrin signaling via the focal adhesion kinase (FAK) - p130Cas pathway, and selectively inhibits integrin-mediated migration of breast cancer cells [9]. To further elucidate the mechanisms of LOX-PP action, co-affinity-purification/mass spectrometry was performed and the “Cbl-interacting protein of 85-kDa” (CIN85) [10] identified as an associating protein.

CIN85 belongs to a small family of adapter proteins that function as docking partners for numerous signaling proteins frequently upregulated in breast cancer [11]. CIN85 and its closely related family member CD2AP share an identical overall domain structure [12]. The CIN85 protein is composed of three amino-terminal Src homology 3 (SH3) domains, followed by a proline-rich (PR) region, which provides binding sites for SH3 domain-containing proteins, an unstructured region of approximately 160 residues, and a carboxy-terminal coiled-coil (CC) domain that can form heterotypic interactions with CD2AP [13]. The CIN85 SH3 domains share similarities among themselves and between family members with overlapping functions identified, e.g., regulation of signaling pathways such as phosphatidylinositol 3-kinase, and Ras GTPase activating protein [12], [14]. The majority of SH3 domains studied so far recognize proline-rich sequences with a minimal core PxxP consensus sequence [15], [16]. Peptide binding can occur in one of two opposite orientations guided by a positively charged residue (+xxPxxP and xPxxPx+, class I and class II respectively) [17] and commonly follows a 1∶1 stoichiometry. The SH3 domains of CIN85 display a novel binding consensus sequence - preferring a PxxxPR peptide binding motif [18]. Many of the SH3 domain interacting proteins are involved in the regulation of cytoskeletal and membrane structures, which also play functional roles in cancer cell invasion [11]. While all three SH3 domains of CIN85 interact with c-Cbl, the SH3-B domain was identified as the major player with ability to enhance the binding of the SH3-A and SH3-C domains with c-Cbl [10]. This observation might be related to the SH3-B domain displaying the highest binding affinity compared to the other two SH3 domains [18]. Notably, elevated CIN85 expression has been associated with the invasive phenotype of triple negative MDA-MB-231 breast cancer cells by regulating the c-Cbl directed mono-ubiquitination of AMAP1 [19]. Here we report that the CIN85 protein is a direct binding partner of LOX-PP, and that their interaction occurs via an atypical ligand motif in LOX-PP and the SH3-B domain of CIN85, as judged by alanine scanning mutations and structural modeling. The interaction with LOX-PP competed for CIN85 association with c-Cbl and profoundly reduced the invasive phenotype of breast cancer cells, identifying a new mechanism to inhibit invasion.

## Materials and Methods

### Plasmids, viral vectors and siRNA

pEGFP-C1-mouse CIN85 and pcDNA-HA-mCIN85 were kindly supplied by Dr. Kan Liao (Shanghai Institutes for Biological Sciences, China) [20]. pcDNA4-rat CIN85 (SETA123cc), and the SH3-A, SH3-B and SH3-C domains, all within pGEX-KG vectors, were kindly supplied by Dr. Oliver Bogler (University of Texas MD Anderson Cancer Center, Houston, TX) [21]. The cDNA encoding rCIN85 WT was digested with *Eco*RI and subcloned into pGEX4T-3 (GE Healthcare, Uppsala, Sweden) and pFLAG-CMV-2 (Sigma-Aldrich, St. Louis, MO). The FLAG-tagged AMAP-1 construct subcloned into pcDNA3 [19] was generously provided by Hisataka Sabe (Hokkaido University School of Medicine, Sapporo, Japan). The HA-ubiquitin construct in pCMV6 was kindly provided by Xaralabos Varelas (Boston University School of Medicine, Boston MA) [22]. To construct CIN85 NT (3 SH3 domains) mutant, the cDNA of rat CIN85 was digested with Kpn*I* and subcloned into pFLAG-CMV2. pFLAG-CD2AP-WT was previously described [13]. pEBG-GST mammalian expression vector was a generous gift of Bruce Mayer (University of Connecticut Health Center, Farmington, CT) [23]. For preparation of recombinant myc-His tagged rat LOX-PP (rLOX-PP-myc-His protein), the signal peptide of Pro-LOX was replaced with the one from osteonectin (BM-40) in the pcDNA4/TO/myc-His vector, as described previously [24]. For construction of C-terminally GST-tagged LOX-PP, the cDNA encoding GST and LOX-PP-WT (1-162 aa), or deletion constructs containing amino acids 1–151, 1–137, 1–128, 1–120, 1–115 or 1–100 were amplified and inserted into pcDNA3.1(+) (Invitrogen, Carlsbad, CA). To generate mutants in which individual amino acid residues (P111 to Q120) of LOX-PP were replaced with alanine (P111A, R112A, P113A, R116A, H117A, W118A, F119A and Q120A), oligonucleotide-directed mutagenesis with Pfu polymerase (Stratagene) was carried out using LOX-PP-1aa-Fw, 5′-cccggatccaccatgcgtttcgcctgg-3′, and LOX-PP-162aa-Rv, 5′-gggatcgatgcccaccatgcgatc-3′ and corresponding primers sets as follow: R110A-Fw, 5′-gtcgccgcgggtgccccccggcccgcc-3′, R110A-Rv, 5′-ggcgggccggggggcacccgcggcgac-3′, P111A-Fw, 5′-gccgcgggtcgtgcccggcccgccgcc-3′ P111A-Rv, 5′-ggcggcgggccgggcacgacccgcggc-3′, R112A-Fw, 5′-gcgggtcgtcccgcccccgccgcccgc-3′, R112A-Rv, 5′-gcgggcggcgggggcgggacgacccgc-3′, P113A-Fw, 5′-ggtcgtccccgggccgccgcccgccac-3′, P113A-Rv, 5′-gtggcgggcggcggcccggggacgacc-3′, R116A-Fw, 5′-cggcccgccgccgcccactggttccaa-3′, R116A-Rv, 5′-ttggaaccagtgggcggcggcgggccg-3′, H117A-Fw, 5′-ccgccgcccgcgcctggttccaagct-3′, H117A-Rv, 5′-agcttggaaccaggcgcgggcggcgg-3′, W118A-Fw, 5′-gccgcccgccacgcgttccaagctggtt-3′, W118A-Rv, 5′-aaccagcttggaacgcgtggcgggcggc-3′, F119A-Fw, 5′-gcccgccactgggcccaagctggtttct-3′, F119A-Rv, 5′-agaaaccagcttgggcccagtggcgggc-3′, Q120A-Fw, 5′-cactggttcgccgctggtttctcgcc-3′, Q120A-Rv, 5′-cagcggcgaaccagtggcgggcggc-3′. Mutations were verified by DNA sequencing. The cDNA of LOX-PP-WT, -P111A and -R116A were inserted into pcDNA4/V5-His vector (Invitrogen). The cDNA of LOX-PP-WT, -P111A and R116A fused with V5-His were then amplified with *Bam*HI and *Not*I and subcloned into pCX_bsr_ (kindly provided by Tsuyoshi Akagi, KAN, Kobe, Japan). The siRNA used for targeting human and mouse CIN85 (SI00141316, Oligonucleotide, 5′-AAGACTGTTACCATATCCCAA-3′) and AllStar negative control siRNA were purchased from QIAGEN (Maryland, MD).

### Cell culture conditions

ER-positive MCF-7, ZR-75, T47D and BT474 cells, and ER-negative Hs578T and MDA-MB-231 cells were purchased from the American Type Culture Collection (ATCC, Manassas, VA) and maintained in culture medium as recommended by ATCC. Trastuzumab-resistant human BT474 breast cancer clone E isolated in the continuous presence of 1 µmol/L trastuzumab during a 5-month selection period, was kindly provided by Susan Kane (Beckman Research Institute of the City of Hope, Duarte, CA), and cultured as described previously [25]. The NF639 cell line, kindly provided by Philip Leder (Harvard Medical School, Boston MA) was derived from a mammary tumor in a mammary tumor virus (MMTV)-*ERBB2* transgenic mouse and cultured as described [5]. The MDA-MB-231 lung metastatic derivative LM2 cell line, kindly provided by Joan Massague (Memorial Sloan-Kettering Cancer Center, NY), was cultured as described [26]. The untransformed mouse mammary epithelial NMuMG cell line was cultured as described previously [27]. Human mammary epithelial MCF10A cells were maintained in 1∶1 mixture of Dulbecco's modified Eagle's medium (DMEM) and Ham's F12 medium with reduced 0.04 mM Ca^2+^ (Invitrogen), 20 ng/ml epidermal growth factor (Sigma-Aldrich), 100 ng/ml cholera toxin (Sigma-Aldrich), 10 µg/ml insulin (Sigma-Aldrich), 500 ng/ml (95%) hydrocortisone (Sigma-Aldrich) and 5% of Chelex-treated horse serum (Invitrogen). Human embryonic kidney HEK293T cells and Bosc23 cells were obtained from the ATCC. These two cell lines were maintained in Dulbecco's minimal essential medium (DMEM) supplemented with 10% heat-inactivated fetal bovine serum (FBS), 2 mM glutamine, and penicillin/streptomycin.

### Transfection and infection of cells in culture

To prepare recombinant retrovirus, pCLEco vector encoding viral component genes (Imgenex, San Diego, CA) was cotransfected with pCX_bsr_ or vector bearing the LOX-PP fragment with C-terminal V5/His tag into the ecotropic packaging cell line Bosc23. After 48 h, NF639 cells were infected with filtered culture supernatant from Bosc23 cells containing viruses, supplemented with 8 µg/ml polybrene (Sigma-Aldrich). Infected cells were selected with 10 µg/ml blasticidin S (Invitrogen) to generate separate pools of stable infectants of EV-, LOX-PP-WT, LOX-PP-P111A and LOX-PP-R116A expressing NF639 cells. For knockdown experiments, cells were transfected at a final concentration of 20 nM using Lipofectamine RNAiMAX (Invitrogen) according to the manufacturer's protocol.

### Affinity isolation, gel electrophoresis and mass spectrometric analysis of LOX-PP-interacting proteins

ZR-75 breast cancer cells, in six 35-mm dishes, were transfected with 1 µg of parental pEBG-GST or mLOX-PP-GST expression plasmid per dish using LipofectAMINE 2000 reagent (Invitrogen) according to the manufacturer's protocol. After 24 h, cells were lysed in 300 µl/dish of Buffer A (25 mM HEPES-KOH (pH 7.2), 150 mM KCl, 2 mM EDTA, 1 mM phenylmethylsulfonyl fluoride, 1 mM dithiothreitol, 0.5 µg/ml leupeptin, 2 µM pepstatin A, 1 µg/ml aprotinin, and 1% Triton X-100 (TX-100)). The lysates were centrifuged at 16,440 x *g* for 10 min at 4°C to remove insoluble material. The supernatant (ca. 2 mg) was incubated with 20 µl Glutathione-Sepharose 4B beads (GE Healthcare) for 2 h at 4°C. The resin was washed four times with lysis buffer and proteins eluted with SDS-PAGE loading buffer. Following separation by 10% SDS-PAGE, proteins were visualized by ProteoSilver Plus Silver Staining Kit (Sigma-Aldrich). Bands corresponding to various molecular weights, including 85 kDa, were isolated. In-gel proteolytic digestion and mass spectrometry (LC/MS/MS) was performed by Taplin Biological Mass Spectrometry Facility (Boston, MA).

### Preparation of recombinant proteins and GST-pull down assays

Purified rLOX-PP-myc-His protein was prepared as described previously [20]. GST and GST-CIN85 WT in the pGEX-4T3, and GST-SH3-A, GST-SH3-B, and GST-SH3-C in the pGEX-KG vector were expressed in *E. coli* BL21 (DE3) pLysS cells (Invitrogen). Bacteria were grown and protein expression induced as described previously [28]. At 3 h post-induction, bacteria were harvested by centrifugation (3,700 x g for 10 min at 4°C), resuspended in Lysis buffer (1% TX-100, 50 mM Tris, 150 mM NaCl, 1 mM EDTA, and 1 mM PMSF (pH 8.0)). Following sonication, the debris was removed by centrifugation at 22,100 x *g* for 30 min. The supernatant was loaded on a Glutathione-Sepharose 4B column, washed extensively with Lysis buffer containing 300 mM NaCl and 0.1% TX-100, and eluted with phosphate buffer (36 mM Na_2_HPO_4_ and 14 mM NaH_2_PO_4_ (pH 7.2)) containing 100 mM NaCl, 0.1% TX-100 and 30 mM glutathione. Binding assays were conducted in phosphate buffer containing 100 mM NaCl and 0.1% TX-100. Purified binding partners, at the indicated concentrations, were incubated for 3 h at 4°C. Glutathione-Sepharose 4B was added and the mixture incubated for 1.5 h at 4°C with gentle rotation. The beads were sedimented in a microcentrifuge. After extensive washing, sample buffer was added and eluted proteins and samples of lysates (4 or 5%) subjected to SDS-PAGE followed by Western blotting (WB), which was performed as we have described previously [3], [28].

### Antibodies

Antibodies from Santa Cruz Biotechnology (Santa Cruz, CA) included GST (B-14), CD2AP (H-290), and CD2AP (B-4), normal rabbit IgG (sc-2027), and normal mouse IgG (sc-2025). Antibodies against CIN85 were from Upstate Biotechnology (Lake Placid, NY) (clone 84) and Calbiochem (Billerica, MA) (#231006). Antibodies against c-Raf (clone 53), c-Cbl (clone 17) and p130Cas (clone 21) were from BD Biosciences. Antibodies against His-tag (#120-003-812), ASAP1 (#600-401-911), and EGFR (#2232) were from Macs Miltenyi Biotec (Germany), Rockland Immunochemicals (Gilbertsville, PA), and Cell Signaling, (Danvers, MA), respectively. Antibodies against HA-tag (12CA5) and HA-HRP (Clone 3F10) were from Roche (Indianapolis, IN), and those against β-actin (AC-15) and FLAG (M2) were from Sigma-Aldrich. Antibodies against GFP (A-6455) and V5 (R960-25) were purchased from Invitrogen. Rabbit polyclonal antibodies against LOX-PP were prepared as described previously [29] and LOX-propeptide antibody (NBP1-30327) was purchased from Novus Biologics (Littleton, CO).

### Immunoprecipitation analysis

Hs578T, MCF-7, ZR-75 or HEK293T cells were lysed with Buffer A, described above. Rabbit antibody against LOX-PP [29], CIN85 or FLAG protein (2 µg) was added to 500 µg cell lysate. After overnight incubation at 4°C, Protein G-Sepharose beads (Invitrogen) were added. The mixture was incubated at 4°C for 2 h with gentle shaking. Beads were then washed four times with Buffer A. The immune-complexes were eluted with SDS-PAGE sample buffer, and the precipitated proteins analyzed by WB. As the band of precipitated LOX-PP migrated close to that of rabbit IgG light chain, Protein A-conjugated HRP was used as a secondary ‘antibody’ to detect immunoprecipitated LOX-PP.

### Ubiquitination assay

The ubiquitination assay was performed essentially as described by Nam et al. [19]. Briefly, HEK293T cells were transiently transfected with the indicated constructs, and after 48 h lysed with RIPA buffer. Immunoprecipitation of FLAG-tagged AMAP-1 was performed with a FLAG specific antibody coupled to Protein-G Sepharose. Proteins retained on beads were subjected to WB with anti-HA-HRP Clone 3F10 antibody to detect HA-tagged ubiquitin. Protein expression of all transfected cDNAs was monitored by WB of total whole cell extracts (30 µg) with Cbl, CIN85 and LOX-PP antibodies as well as FLAG antibody for AMAP-1.

### Structural modeling

Firstly, a comparative model of the mouse CIN85 (SH3KBP1) SH3-B domain (Uniprot code: Q8R550, amino acids 101-155) was prepared using *Modeller 9* [30]. The closest matching sequence from structures deposited in the Protein Data Bank (PDB) was determined by *Modeller 9*'s dynamic programming algorithm. After excluding existing NMR structures of the same domain, as they diverge from the expected conformation of a binary complex, the most similar template was the crystal structure with PDB code 3U23, which has 71% sequence identity. This is a binary complex of the SH3-B domain from a related protein, CD2AP, and a proline-rich epitope from Rin3. Comparative modeling resulted in 5 similar structures, and the model ranking with the lowest score of the modeler objective function was selected. Crystal structures of related SH3-domain binary complexes bound to peptides harboring similar PxxxxR motifs (3U23, 2BZ8, 2DF6) were superimposed on the model generated. Using this structural alignment as a guide due to significant overlap in their backbone coordinates, a peptide chain corresponding to the mouse LOX-PP epitope region (^110^RPRPAARHW^118^) could be manually built and docked to the CIN85 SH3-B surface within *COOT* [31] ensuring contacts between residues of the interacting motif and the SH3 surface. Finally, energy minimization was carried out in an explicit solvent shell using the *YASARA* force field, which is an improved approach that combines the *AMBER* all-atom force field with multi-dimensional knowledge-based torsional potentials, implemented at www.yasara.org/minimizationserver [32].

### Collagen degradation assay

Circular glass coverslips (15-mm, Fisher) were coated with 20 µg/ml FITC-conjugated gelatin (Invitrogen) suspended in 20 mg/mL sucrose in PBS for 1 h. The gelatin solution was removed, and the protein crosslinked with addition of 0.5% glutaraldehyde in PBS for 15 min. After extensive washing with PBS, the reaction was quenched with 5 mg/ml sodium borohydride for 3 min, followed with several washes in PBS. NF639 or MDA-MB-231 cells, treated with siRNAs, were plated on the gelatin-coated coverslips and cultured in DMEM supplemented with media containing 10% heat-inactivated FBS for 4 h. To assess for gelatin degradation, cells were fixed with 4% paraformaldehyde in PBS, treated with 0.2% TX-100 in PBS, and incubated with 2% bovine serum albumin in PBS. The coverslips were incubated with TRITC-phalloidin (Cytoskeleton, Denver, CO). Subsequently, the coverslips were mounted in Slowfade Gold antifade reagent (Invitrogen) with Hoechst 33342 (Invitrogen). Fluorescence microscopy was performed with a Nikon Eclipse E400 microscope with a 50× objective lens. Confocal microscopy was performed with a Zeiss LSM 510 200M confocal microscope with a 63× lens (Thornwood, NY). To quantify the effects of siRNA or LOX-PP on matrix degradation, the percent cells with contact points displaying collagen degradation was determined as follows. The number of cells within a random field with degraded FITC-gelatin was counted and given as percent of the total cell number determined as Hoechst 33342 positive cells. A total of 7-10 fields per experiment were counted from three independent experiments performed in duplicate. Values are given as average percent +/- SD.

### Invasion assays

Polycarbonate filters (6.5 mm, 8 µm pore size) were precoated with 5 µg of collagen type I (BD Biosciences) and placed in the upper chamber of Costar Transwells (Corning, Lowell, MA). NF639 cells (1×10^4^) were suspended in serum-free medium and layered onto the filters, in triplicate, and incubated at 37°C. After 6 h, cells were fixed with 100% methanol. The cells retained in the upper chamber were scraped off with a cotton swab and cells that had invaded to the lower side were stained with crystal violet and counted using a microscope. The average migration from three independent experiments ± SD is presented relative to the control EV, which was set at 100%. *P* values were calculated using a Student's *t*-test.

For Matrigel outgrowth assays, 5×10^3^ NF639 cells per well were seeded on eight-well glass bottom-chamber slides coated with 6 mg/ml Matrigel (BD Biosciences, San Jose, CA), cultured in DMEM supplemented with 10% heat-inactivated FBS, 2 mM glutamine, and penicillin/streptomycin. After 5 days, slides were photographed at 40× magnification.

## Results

### Affinity isolation identified CIN85 as a novel LOX-PP-interacting protein

**Figure 1:**
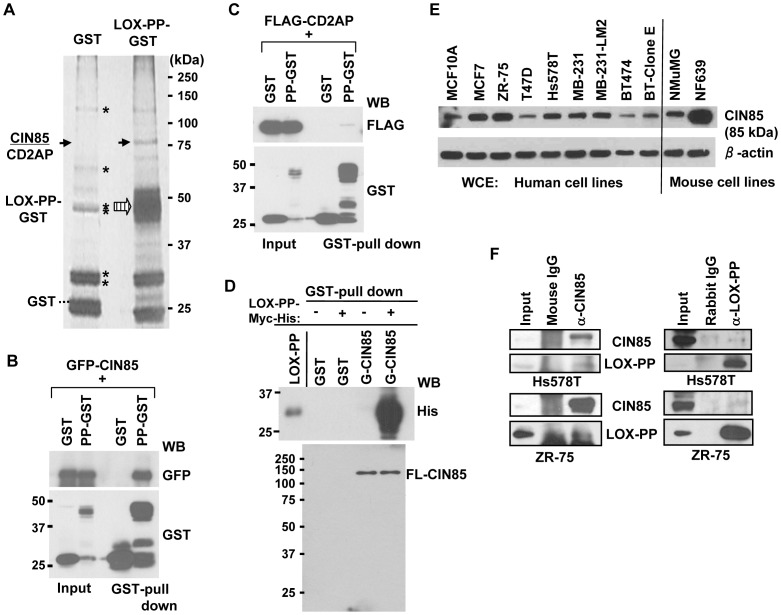
Identification of CIN85 and CD2AP as LOX-PP interacting proteins in breast cancer cells. (**A**) Extracts from ZR-75 cells transfected with vectors expressing GST or LOX-PP-GST were precipitated with Glutathione-Sepharose 4B beads, resolved by SDS-PAGE and silver stained. The band(s) at ∼85 kDa was analyzed by LC-MS/MS mass spectrometry and identified as CIN85 and CD2AP. *, non-specific proteins. The positions of the co-precipitated CD2AP/CIN85 and LOX-PP proteins are indicated by the solid and large hatched arrows, respectively, and of GST by the dashed line. (**B–C**) GST or LOX-PP-GST (PP-GST) was co-expressed with GFP-CIN85 WT (B) or FLAG-CD2AP (C) in HEK293T cells, and LOX-PP associated proteins isolated by GST-pull down assays and subjected to WB for GFP (B) or FLAG (C) and GST. Input, 4% of lysates (4%). (**D**) Recombinant LOX-PP-myc-His (0.5 µM) was subjected to a GST-pull down assay using 0.5 µM of either GST or GST (G)-CIN85, and WB for the His or CIN85 (Calbiochem) antibody. Input, 5%. (**E**) Samples of whole cell extracts (10 µg) of the indicated human and mouse cells were subjected to WB for CIN85 (Upstate). (**F**) (Left) TX-100 extracts of Hs578T (Upper) or ZR-75 (Lower) cells were immunoprecipitated with mouse IgG or CIN85 (Upstate) antibody, and analyzed for CIN85 (Upstate) and LOX-PP. (Right) TX-100 extracts of Hs578T (upper) or ZR-75 (lower) cells were immunoprecipitated with rabbit IgG or LOX-PP antibodies, and subjected to WB.

To identify proteins that associate with LOX-PP in breast cancer cells, GST-tagged murine LOX-PP or control vector DNA (pEBG, encoding GST tag only) was expressed in ZR-75 cells for 24 h. Extracts were incubated with Glutathione-Sepharose 4B resin, and bound proteins washed extensively and eluted with SDS-PAGE loading buffer. Following SDS-PAGE, proteins were visualized by silver staining (Fig. 1A). Bands of approximately 85 kDa were seen in LOX-PP-GST precipitates and not in the control GST lane. These were excised and subjected to in-gel proteolytic digestion and mass spectrometry (LC/MS/MS). The bands were identified as the 85-kDa cytoplasmic adapter protein CIN85 (UniProtKB accession number: Q96b97) and as CD2AP (accession number: Q9y5k6). In total, 22 and 42 peptides were identified for CIN85 and CD2AP, respectively, resulting in coverage of 35.8% of amino acids (aa) (238/665) and 49.8% of aa (318/639) (Figure S1). To confirm the mass spectrometry analysis, the GST-LOX-PP and GST proteins were co-expressed with either GFP-CIN85 or FLAG-CD2AP in HEK293T cells and a small-scale GST-pull down assay performed (Fig. 1B, C). Both GFP-CIN85 and FLAG-CD2AP specifically co-purified with GST-LOX-PP vs. GST, although the binding of LOX-PP to CIN85 appeared substantially stronger than to CD2AP. Interestingly, endogenous CD2AP was co-precipitated more efficiently in the presence of GFP-CIN85 (Figure S2), suggesting CIN85 may be an intermediate between LOX-PP and CD2AP. Thus, we next asked whether CIN85 can interact directly with LOX-PP. Using purified preparations of myc-His-tagged rLOX-PP and GST-CIN85 protein, CIN85 protein was shown to effectively bring down recombinant LOX-PP, confirming the direct association between these two proteins (Fig. 1D). To evaluate levels of CIN85 expression in breast cancer cells, whole cell extracts were prepared from two untransformed lines: human MCF10A and mouse NMuMG cells and from nine breast cancer cell lines: human MCF7, ZR-75, T47D, Hs578T, MDA-MB-231, MDA-MB-231-derived LM2, BT474, and trastuzumab-resistant BT474-Clone E cells, and murine NF639. Western blot analysis showed higher levels of CIN85 protein in most of the breast cancer lines compared to the untransformed MCF10A and NMuMG cells (Fig. 1E). We next tested for association between endogenous LOX-PP and CIN85 in breast cancer cells and selected the Hs578T and ZR-75 lines, which expressed a relatively high level of CIN85. Triton X-100 extracts were prepared from both lines and incubated with an antibody against CIN85 or control mouse IgG (Fig. 1F, left panels) or against LOX-PP or control rabbit IgG (Fig. 1F, right panels). In both lines, the endogenous LOX-PP and CIN85 proteins co-precipitated with CIN85 and LOX-PP antibodies, respectively (Fig. 1F). The appropriate co-precipitating bands were not seen with the control IgG antibodies, although a strong background smear was observed with the mouse IgG control. Taken together, these studies indicate that LOX-PP can directly associate with CIN85 in breast cancer cells.

### LOX-PP competes via an atypical ligand with c-Cbl for interaction with the SH3-B domain of CIN85

**Figure 2:**
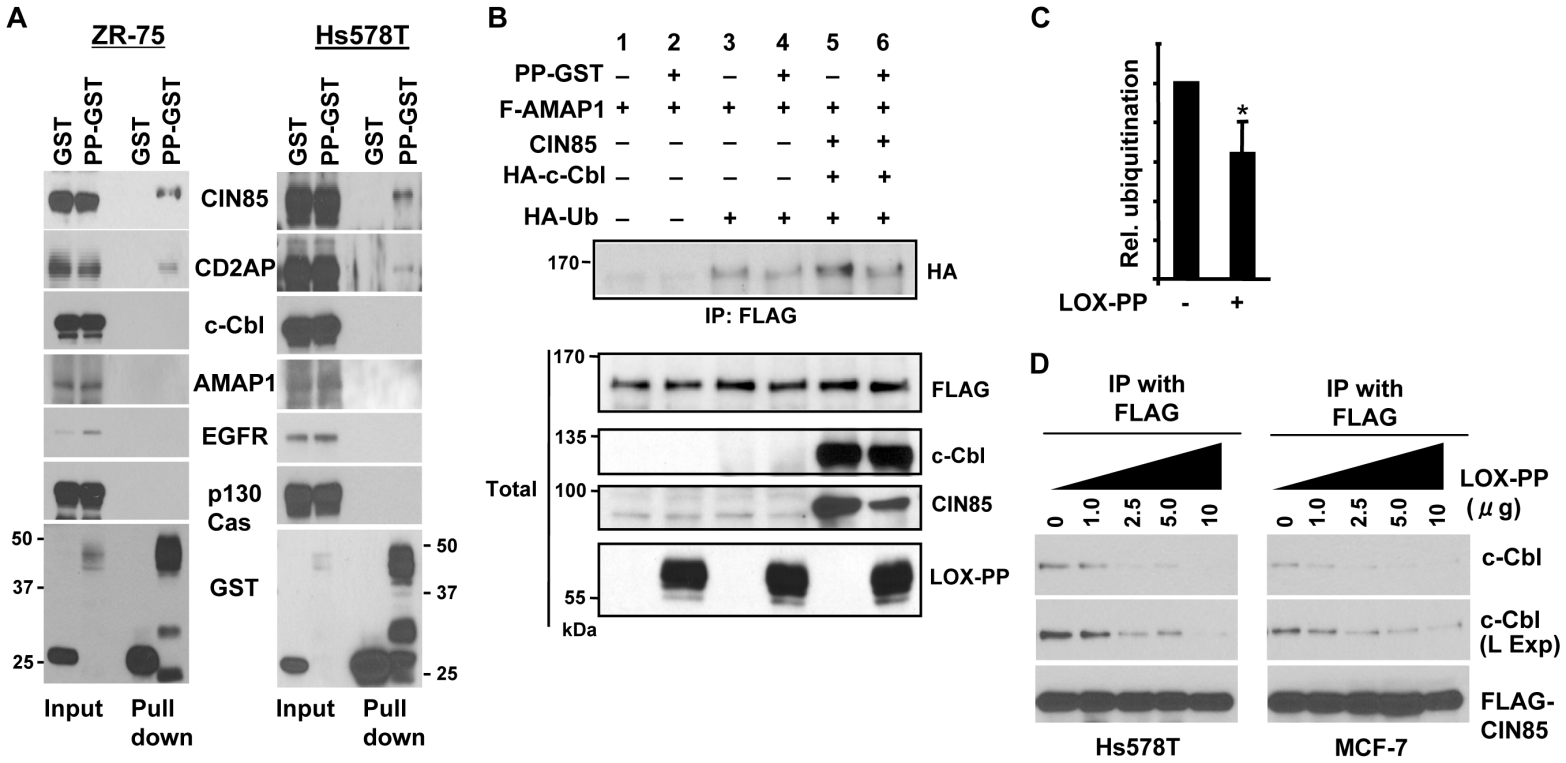
LOX-PP reduces CIN85 mono-ubiquitination and ability to interact with c-Cbl. (**A**) GST or LOX-PP-GST (PP-GST) was expressed in ZR-75 (left panel) or Hs578T (right panel) cells. GST and associated proteins were precipitated as described in Fig. 1B and subjected to WB for CIN85 (Upstate Biotechnology and Calbiochem antibodies for ZR-75 and Hs578T cells, respectively), CD2AP (H-290), c-Cbl, AMAP1, EGFR, p130Cas and GST. Input, 4%. (**B–C**) HEK293T cells were transfected with AMAP1-FLAG, CIN85, HA-c-Cbl, HA-ubiquitin and LOX-PP-GST (PP-GST) as indicated and subjected to a ubiquitination assay. FLAG-tagged AMAP1 was immunoprecipitated and total whole cell extracts were subjected to WB with an HA antibody (upper panel), or the indicated antibodies (lower panel). (B). Data were quantified and relative mono-ubiquitination of AMAP1 with and without LOX-PP was determined by averaging the results of three independent experiments (C). *P* value was calculated using Student's *t*-test. *, P<0.03. (**D**) FLAG-CIN85 was expressed in Hs578T (left panel) or MCF-7 (right panel) cells. After lysis, the indicated amount of recombinant LOX-PP-myc-His was added and the mixture incubated at 4°C for 2 h. Proteins were then immunoprecipitated with a FLAG antibody and subjected to WB with FLAG and c-Cbl antibodies. (L Exp, longer exposure).

To begin to address the functional role of the LOX-PP interaction with CIN85, we tested for the ability of LOX-PP to associate with CIN85-interacting proteins. Ectopically expressed LOX-PP-GST in ZR-75 and Hs578T cells brought down CIN85 and CD2AP. No association of LOX-PP was detected with the CIN85-interacting proteins c-Cbl, ASAP1/AMAP1, epidermal growth factor receptor (EGFR), or p130Cas (Fig. 2A), or with sprouty2, Src, Ras or SHIP1 (data not shown). The CIN85 interacting protein AMAP1 has been associated with tumor invasion and in particular when it is mono-ubiquitinated by c-Cbl in complex with CIN85 [33]. LOX-PP was shown to significantly attenuate the mono-ubiquitination of AMAP1 (Fig. 2B). Analysis of three independent experiments indicated LOX-PP reduced AMAP1 mono-ubiquitination by ∼36% (Fig. 2C).

The failure to detect c-Cbl in the LOX-PP/CIN85 complex was unexpected. This did not appear to relate to an inability of CIN85 to interact with c-Cbl in breast cancer cells as this association was confirmed by successful co-immunoprecipitation of the two proteins in lysates from Hs578T, MCF7 and ZR-75 cells (data not shown). This led us to hypothesize that the interaction of CIN85 and LOX-PP could be mediated via the same SH3 domain(s) as with c-Cbl, in which case LOX-PP might be more effectively binding and therefore competing for CIN85 binding. We tested this possibility using a competition assay (Fig. 2D). An increasing amount of purified LOX-PP was added to immunoprecipitates of Hs578T and MCF7 cell lysates overexpressing FLAG-tagged CIN85. LOX-PP efficiently displaced endogenous c-Cbl from CIN85 (Fig. 2D), suggesting LOX-PP was able to compete for binding.

**Figure 3:**
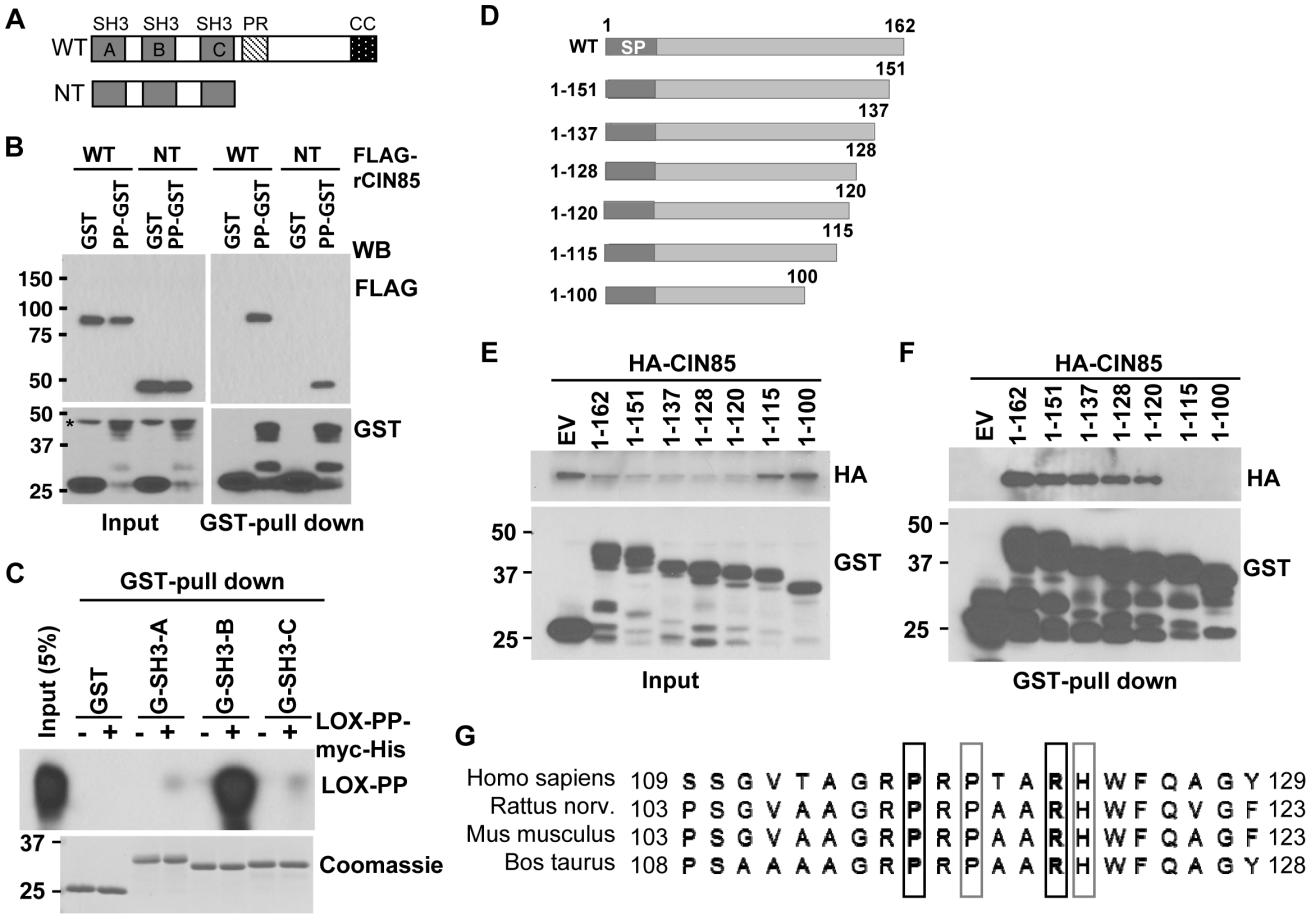
The SH3-B domain of CIN85 interacts with the LOX-PP region preceding aa 120. (**A**) Schematic representation of the full length CIN85 (WT) and the amino terminal-containing deletion mutant (NT). The SH3, Src homology 3 domains A, B, and C, PR, proline-rich region, CC, coiled-coil domain are indicated. (**B**) GST or LOX-PP-GST (PP-GST) protein was co-expressed with FLAG-tagged CIN85 WT or CIN85 NT in HEK293T cells. GST-pull down assays were performed and bound or whole cell extracts subjected to WB with FLAG and GST antibodies. (**C**) Recombinant LOX-PP-myc-His (0.5 µM) was incubated with GST, GST (G-)-tagged SH3-A, SH3-B or SH3-C peptides (0.5 µM each) and subjected to a GST-pull down assay. The precipitated proteins were analyzed by Coomassie staining (lower panel) and with antibodies against LOX-PP (upper panel). (**D**) Schematic representation of LOX-PP deletion mutants prepared in the pcDNA3-GST vector. SP, Signal peptide. The lengths of the constructs are indicated on the left. (**E–F**) HA-CIN85 was co-transfected with either LOX-PP-WT (1-162) or the indicated deletion mutants (from part D) or empty vector (EV) DNA into HEK293T cells. Extracts were prepared and samples (4%) were analyzed directly as a measure of input (E) or subjected to GST-pull down assays and WB for HA and GST (F). (**G**) Amino acid sequences of LOX-PP from various species are shown. The positions of aa 111 and aa 116 and of aa 113 and aa 117 are indicated by black and grey boxes, respectively.

To test for the role of the SH3 domains in the interaction of LOX-PP and CIN85, a construct expressing the three SH3 domains (NT) was prepared (Fig. 3A). Full-length CIN85 and the NT construct were both found to effectively interact with LOX-PP in GST-pull down experiments (Fig. 3B), suggesting the interaction was indeed mediated by one or more SH3 domain. To address this question, the individual domain(s) of CIN85 were cloned, GST-tagged recombinant SH3 domain peptides were prepared and used in pull down assays to test separately which one(s) is mediating interaction with LOX-PP (Fig. 3C). GST-SH3-B, but not GST-SH3-A or GST-SH3-C, effectively brought down recombinant LOX-PP, suggesting the direct association between these two proteins occurs via the SH3-B domain. To begin to map the region(s) of LOX-PP mediating binding with CIN85, a series of progressive C-terminal deletion mutants were constructed (Fig. 3D). LOX-PP-GST, deletion mutant, or empty vector GST proteins were co-expressed with HA-CIN85 in HEK293T cells and GST-pull down assays performed (Fig. 3E, F). As LOX-PP is a secreted protein with a signal peptide and able to be taken up into cells [5], [9], intracellular LOX-PP was detected. While LOX-PP variant aa 1-120 bound HA-CIN85, the binding was completely lost when aa 115 to 162 of LOX-PP were deleted. Thus, the major CIN85 binding region of LOX-PP is positioned in the region preceding aa 120. Of interest, we noted that this region of LOX-PP does not encode a ‘classical’ PxxP SH3 binding core motif, but instead contains a PxPxxR motif, which is highly conserved in different species (Fig. 3G) and somewhat reminiscent of the PxxPxR core motif reported for some well studied SH3 domains (e.g. the Grb2 SH3-N domain).

### P111 and R116 of LOX-PP are required for binding to CIN85 and for competing with c-Cbl binding

**Figure 4:**
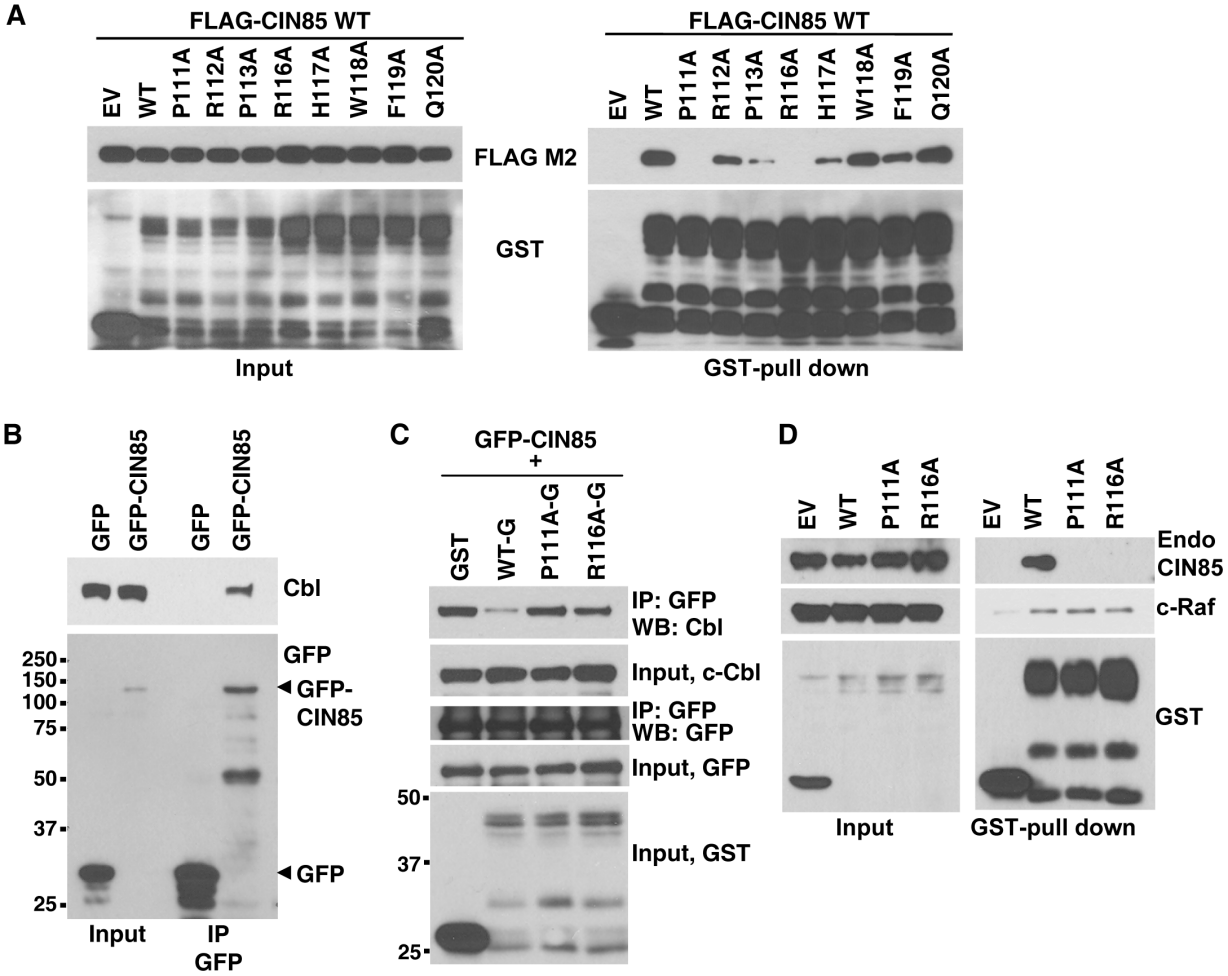
Alanine scanning mutagenesis reveals amino acids of LOX-PP required for CIN85 binding and c-Cbl competition. (**A**) To begin to identify the critical amino acids in LOX-PP, vectors expressing individual point mutants in which residues aa 111 to aa 120 were replaced with alanine, or WT LOX-PP protein were co-transfected with FLAG-CIN85 WT in HEK293T cells. (right panel) The mutated proteins compared with the WT LOX-PP for their ability to interact with CIN85 using GST-pull down assays and WB with FLAG and GST antibodies. (left panel) Input, 4%. EV, empty vector DNA. (**B**) To confirm the interaction of CIN85 and endogenous c-Cbl, GFP or GFP-CIN85 proteins were expressed in HEK293T cells and TX-100 extracts prepared. Following immunoprecipitation with a GFP antibody, the precipitated proteins subjected to WB with antibodies against c-Cbl (upper panel) and GFP (lower panel). (**C**) To test whether LOX-PP mutants unable to interact with CIN85 compete for its binding with c-Cbl, GFP-tagged-full-length CIN85 was co-expressed with GST, or GST-tagged LOX-PP-WT (PP-WT-G), LOX-PP-P111A (PP-P111A-G), or LOX-PP-R116A (PP-R116A-G) in HEK293T cells. TX-100 extracts were immunoprecipitated with GFP antibody and the precipitated proteins detected with antibodies against c-Cbl, GFP and GST. Input, 4%. (**D**) To test the specificity of the changes in binding of the mutant LOX-PP proteins, vectors expressing GST tagged LOX-PP-WT (WT), or mutants LOX-PP-P111A (P111A) or LOX-PP-R116A (R116A) or GST (EV) were transfected into ZR-75 cells and their ability to interact with c-Raf, which maps to aa 26-100, monitored by GST-pull down assays. WB for CIN85 (Upstate: clone 84), c-Raf and GST was performed.

The SH3 domains of CIN85 have been previously reported to bind an unconventional consensus sequence of PxxxPR [9]. To identify the critical residues in LOX-PP, we replaced each amino acid between position 111 to 120 individually with alanine (except for positions 114 and 115, which were already alanines and not mutated) and assayed for binding to FLAG-tagged CIN85. Binding was completely abolished by the P111A and R116A mutations, while more modest effects were seen with the P113A and H117A mutations, suggesting that the P111 and R116 residues contribute substantially to the interaction of LOX-PP with the CIN85 SH3-B domain (Fig. 4A). One would predict that if the LOX-PP mutants are unable to interact with CIN85, then they would fail to compete for binding with c-Cbl. To test this hypothesis competition assays were performed. Specifically, lysates from HEK293T cells overexpressing GFP-tagged CIN85 were immunoprecipitated in the absence (Fig. 4B) or presence (Fig. 4C) of LOX-PP-WT, -P111A or -R116A. LOX-PP-P111A and -R116A were unable to efficiently displace endogenous c-Cbl from GFP-CIN85 in contrast to the effects of LOX-PP-WT (Fig. 4B, C). Lastly, the specificity of the changes in binding due to these mutations in LOX-PP was examined by comparing their effects on interaction with c-Raf, which was previously mapped to aa 26-100 [28]. While LOX-PP mutants P111A and R116A have lost the ability to interact with CIN85, they retained the ability to interact with c-Raf essentially as well as WT LOX-PP (Fig. 4D).

### Modeling of the LOX-PP ligand confirms identities of critical amino acids

**Figure 5:**
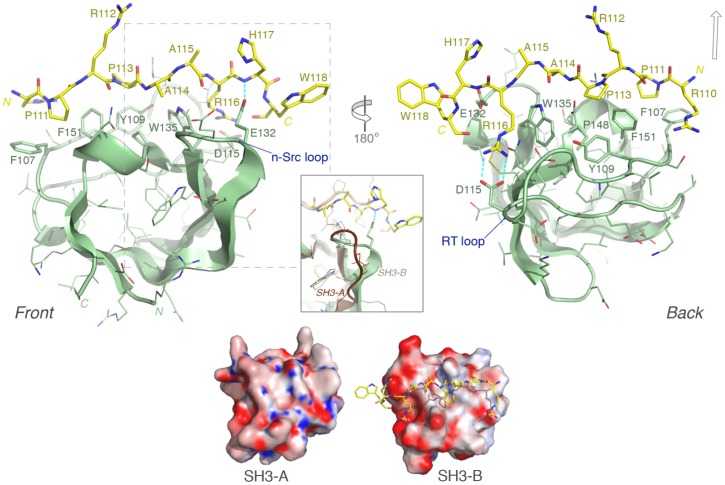
Structural model of CIN85 SH3-B domain in complex with the LOX-PP peptide. *Upper part*: Front and rear views of the modeled SH3-B (pale green) and LOX-PP (yellow sticks) complex are depicted; selected, key electrostatic interactions are represented by blue dashed lines. The open arrow (upper right) indicates the approximate position of Arg(-2) in structures that can assemble the ternary complex: SH3–[RxP^1^xxxxR/K peptide]–SH3. *Inset box*: Detail showing the structural dissimilarity in the n-Src loop between CIN85 SH3-B (our model) and SH3-A, superimposed (PDB code: 2BZ8; brown cartoon). In the SH3-A structure, the bound Cbl-b peptide backbone (light brown) is also seen to diverge more towards its C-terminus from the LOX-PP peptide position. ***Lower part*** **:** Comparison of the electrostatic potential surfaces of SH3 domains A and B in equivalent orientations, illustrating their differential charge distributions. The Adaptive Poisson-Boltzmann Solver (APBS) software [55] was used to generate the electrostatic potential map, contoured in varying colour intensity from -15 (red) through 0 (white) to +15 (blue) kT/e, and rendered within PyMOL (www.pymol.org).

In order to gain additional insights into the interactions between CIN85 and LOX-PP, we generated a molecular model of the CIN85 SH3-B domain in complex with a 9-mer peptide (RPRPAARHW) covering the binding epitope on LOX-PP (Fig. 5, upper panel). The final energy-minimization step resulted in a decrease in the protein model's global energy from −24.7×10^−3^ to −40.8×10^−3^ kJ/mol and a positive quality Z-score of 0.89 (*YASARA* energy terms); 100% of residues lie in the favored regions of the Ramachandran plot. The modeled LOX-PP epitope incorporates a polyproline type-II (PP-II) helix, which in a class II ligand-like orientation enables the intercalation of Pro111^LOX-PP^ and Pro113^LOX-PP^ between predominantly hydrophobic residues (Phe107, Tyr109, Trp135, Pro148 and Phe151) comprising much of the binding groove in CIN85 SH3-B (Fig. 5, upper panel). Furthermore, Arg116^LOX-PP^ forms a salt bridge with Asp115^CIN85^, and the tri-carbon stretch of its side-chain lies nearly parallel to, and makes a hydrophobic contact with, Trp135 ^CIN85^. A similar PP-II conformation and bonding arrangement is also seen, for example, in both CIN85 SH3-A and β-PIX SH3 domain complexes with a Cbl-b peptide [34]. These critical LOX-PP positions (Pro111, Pro113 and Arg116) corroborate the GST-pull down data with alanine mutants seen above in Fig. 4. We note that the H117A^LOX-PP^ mutation also weakened the binding to CIN85, albeit to a lesser extent, and this model suggests that with a subtle reorientation of the C-terminal end of the peptide, His117^LOX-PP^ could form a stabilizing electrostatic interaction with Glu132^CIN85^, hence contributing additional binding energy. The adjacent Trp118^LOX-PP^ appears to be oriented away from the SH3 surface, and thus should play no role in bonding, which reflects the pull down result with the W118A^LOX-PP^ mutant (Fig. 4). Some crystal structures have revealed that ternary complex formation is possible with two SH3 domains docking onto one proline-rich peptide when there is an Arg residue in the -2 position relative to the first Pro (*i.e.* Pro111^LOX-PP^) 34,35. In LOX-PP there is an N-terminal Arg in the -1 position. Its opposite orientation suggests it is therefore unlikely to promote ternary complex formation, at least not in the same manner. Lastly, the interaction between LOX-PP appears to be exclusive to the SH3-B domain (see Fig. 3A). The overlaid surfaces of CIN85 SH3 domains A (crystal structure: 2BZ8) and B (our model) were compared (Fig. 5, lower part). Computed electrostatic potential maps of SH3-A and -B revealed strikingly different charge distributions near to the binding groove in spite of the 47.1% sequence identity (Fig. 5, lower part). This may account in part for the differential binding, but other factors may also play a role (see Discussion). Overall, the structural model fits well with the linker scanning mutational analysis of LOX-PP.

### LOX-PP attenuates invasive phenotype via CIN85 interaction

**Figure 6:**
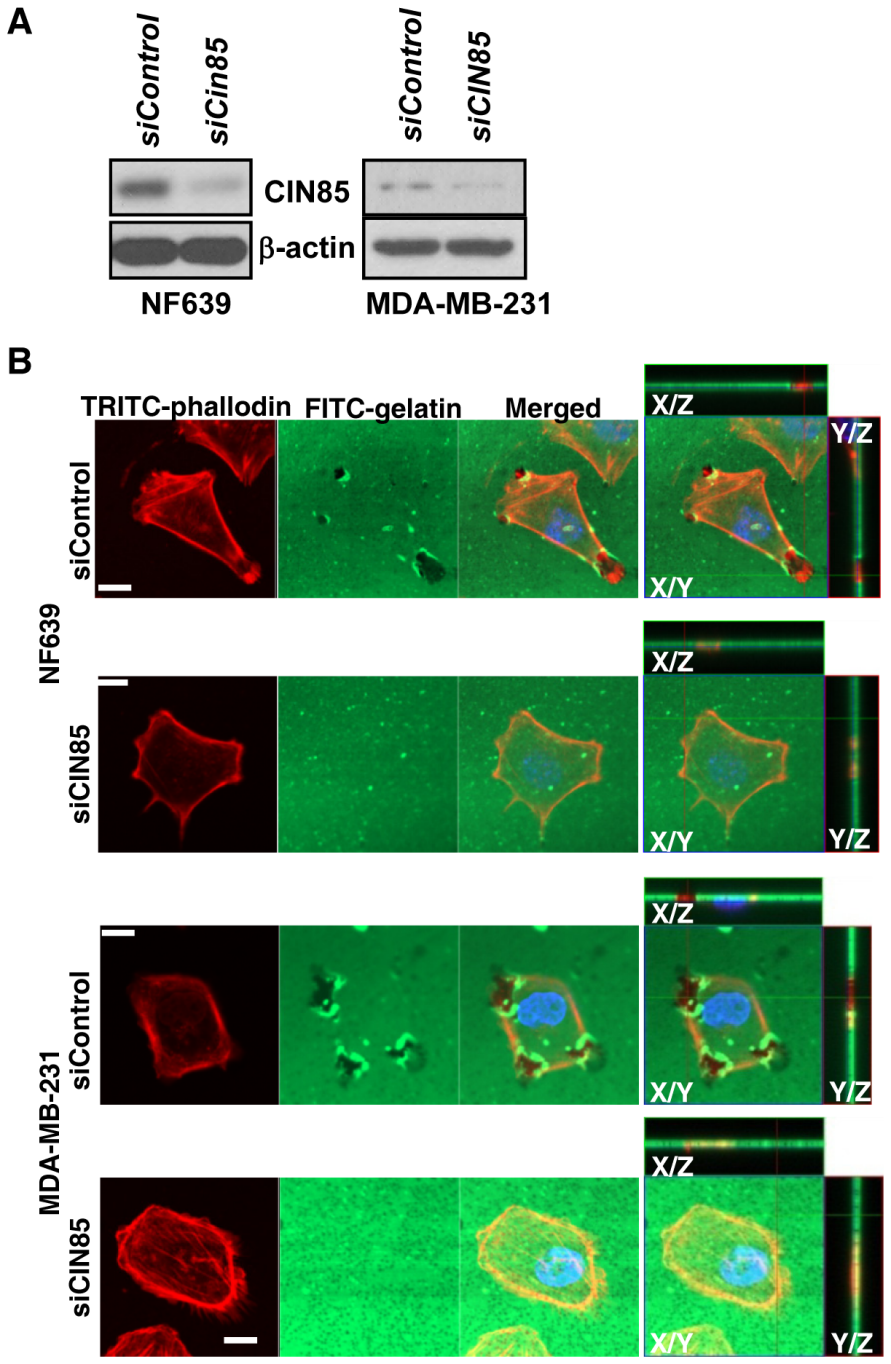
CIN85 promotes degradation of the extracellular matrix. NF639 and MDA-MB-231 breast cancer cells were transfected with 20 nM of either siCIN85 or scrambled negative control siRNA (siControl). (A) After 48 h, samples of whole cell extracts (10 µg) were subjected to WB for CIN85 (Upstate: clone84) and β-actin. (B) Alternatively, cells were plated on coverslips coated with FITC-conjugated gelatin and incubated for 4 h. Cells were fixed 3.7% paraformaldehyde. F-actin and nuclei were labeled with TRITC-phalloidin (red) and Hoechst 33342 (blue), respectively. Lines indicate region of XY image projected to generate orthogonal planes XZ and YZ. Bars: 10 µm.

The CIN85 - c-Cbl complex is recruited to the AMAP1 complex where its ubiquitination activity of c-Cbl is necessary for cancer cells to develop an invasive phenotype [19]. One hallmark of an invasive phenotype is the ability to remodel and degrade the surrounding extracellular matrix at sites of contact [36]. We first tested the effects of CIN85 on collagen degradation by NF639 and MDA-MB-231 cells using FITC-labeled gelatin. Effective knockdown of CIN85 with a specific siRNA vs. siControl was confirmed using WB (Fig. 6A). Following incubation for 4 h, matrix degradation was visualized using confocal microscopy (Fig. 6B). The degradation of the gelatin observed with siControl NF639 and MDA-MB-231 cells was greatly reduced upon knockdown of CIN85. The data from three independent experiments were quantified. While in the siControl cell populations 55.8%±6.2 of NF639 cells and 54.8%±16.9 of MDA-MB-231 cells displayed degraded type I matrix, these numbers were reduced to only 5.6%±2.7 of NF639 cells and 9.1%±3.3 MDA-MB-231 cells degraded upon knockdown of CIN85. These data demonstrate that the degradation of the extracellular matrix by these invasive breast cancer cells is dependent on CIN85.

**Figure 7:**
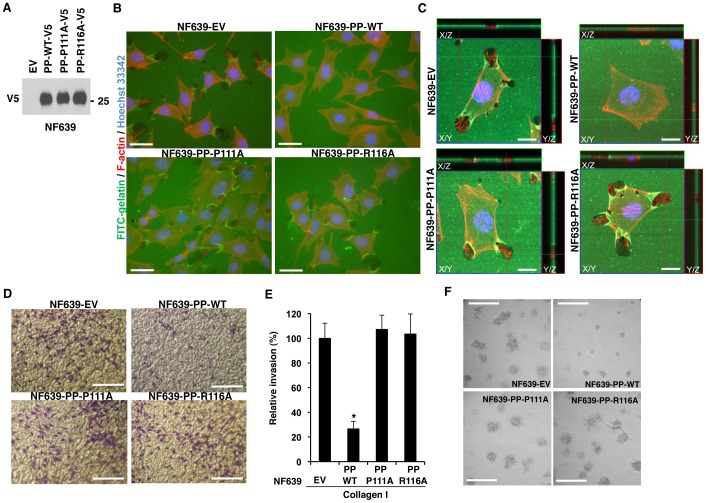
LOX-PP mutants unable to interact with CIN85 are unable to inhibit breast cancer cell invasion. Stable transductants of NF639-EV, and NF639-LOX-PP (PP) -WT, -P111A and -R116A cells were prepared. (**A**) These were analyzed by WB for LOX-PP expression using the V5 tag. (**B,C**) Cells were subjected to a collagen degradation assay using coverslips coated with FITC-conjugated gelatin (green). F-actin and nuclei were labeled with TRITC-phalloidin (red) and Hoechst 33342 (blue), respectively and cells photographed at 50x magnification. Bars: 20 µm (B). Alternatively, confocal images of the slides are shown. Lines indicate region of XY image projected to generate orthogonal planes XZ and YZ. Bars: 10 µm (C). (**D,E**) The indicated NF639 cells were subjected to invasion assays, in triplicate, and photographed (Bar: 0.25 mm) (D). The numbers of cells invaded per field were determined (E).The average data from three independent experiments ± SD is presented relative to the EV control (set at 100%). *P* values were calculated using Student's *t*-test. *, P < 0.01 (E). (**F**) Stable transductants of NF639 cells were subjected to a Matrigel outgrowth assay. After 5 days, slides were photographed at 40x magnification. Bars: 0.25 mm.

We next sought to test the biological consequences of LOX-PP interaction on the ability of CIN85 to promote matrix degradation. We established stable transfected populations of NF639 cell line that express V5-tagged LOX-PP-WT, LOX-PP-P111A, and LOX-PP-R116A, and with the parental empty vector (EV), as control. Equal volumes of media from cultures containing equal numbers of cells were subjected to WB with V5 tag, and approximately equal levels of LOX-PP proteins were seen (Fig. 7A). Cultures of NF639-EV, NF639-LOX-PP-WT, NF639-LOX-PP-P111A and NF639-LOX-PP-R116A cells were plated on the FITC-labeled gelatin and incubated for 4 h. Matrix degradation was visualized using fluorescence microscopy (Fig. 7B) and confocal fluorescence microscopy (Fig. 7C). Ectopic expression of WT LOX-PP reduced the percent of cells degrading matrix with control EV DNA from 75.8%±12.8 to 14.0%±19.7. In contrast, little change in percent of cells degrading gelatin was seen upon expression of either LOX-PP-P111A, or LOX-PP-R116A (70.4%±6.1 and 74.4%±14.9, respectively consistent with their inability to interact with CIN85. Thus, NF639 cell populations stably expressing LOX-PP-WT displayed strongly reduced degradation of ECM proteins compared to EV-control, or cells expressing LOX-PP-P111A or -R116A.

The effects of LOX-PP expression on the ability of NF639 cells to invade through a matrix was measured using a modified Boyden chamber and Matrigel outgrowth assays. NF639 cells stably expressing LOX-PP-WT, LOX-PP-P111A or LOX-PP-R116A or cells transfected with empty vector DNA were tested, in triplicate, for their ability to invade through type I collagen. After 6 h, invaded cells were stained with crystal violet and photographed (Fig. 7D), and the number of cells that had invaded per field was determined in three independent experiments (Fig. 7E). A profound decrease in collagen invasion by breast cancer cells was noted with LOX-PP-WT compared to the control EV cells. In three independent experiments, performed in triplicate, LOX-PP-WT led to a 73.5+/- 5.9% reduction in invasion of NF639. In contrast, no reduction in NF639 cell invasion was seen with the LOX-PP-P111A and LOX-PP-R116A mutants that are unable to interact with CIN85 (Fig. 7E). LOX-PP had no significant effects on cell proliferation over the 6 h time course (data not shown). The effects of LOX-PP on the ability of NF639 cells to form invasive colony outgrowth in Matrigel were also examined. NF639-EV cells formed branching, invasive colonies after 5 days of culture in Matrigel (Fig. 7F). Only small round colonies were observed with NF639-LOX-PP-WT cells, indicating expression of LOX-PP-WT prevented outgrowth. The mutant LOX-PP proteins unable to interact with CIN85 expressed in NF639-LOX-PP-P111A and NF639-LOX-PP-R116A cells were unable to reduce invasive outgrowth in Matrigel. Thus, LOX-PP interaction with CIN85 leads to a decreased invasive phenotype of breast cancer cells.

## Discussion

Here, an atypical proline-rich CIN85 SH3 interacting ligand PxpxxRh was identified in the tumor suppressor protein LOX-PP that functionally inhibits CIN85-mediated invasion by breast cancer cells. Using co-purification/mass spectrometry, CIN85 was identified as a novel interacting partner of the tumor suppressor protein LOX-PP. Their direct interaction was confirmed and the interacting regions mapped to the SH3-B domain of CIN85 and to amino acids 111 to 119 of LOX-PP. Interaction of c-Cbl with CIN85, which is primarily mediated by the CIN85 SH3-B domain, was effectively competed by purified LOX-PP and AMAP1 mono-ubiquitination was attenuated. Using linker scanning mutagenesis and structural modeling, Pro111 and Arg116 were identified as key residues of LOX-PP interaction with CIN85. Consistently mutation of these amino acids decreased the ability of LOX-PP to reduce invasion through type I collagen, and invasive outgrowth in Matrigel and to compete for c-Cbl binding with CIN85. Thus, interaction of the atypical ligand of LOX-PP with CIN85 mediates the ability of this tumor suppressor to reduce invasive phenotype of breast cancer cells. Overall, our findings suggest further analysis of the potential use of this region of LOX-PP in treatment of invasive breast cancers is warranted.

CIN85 was originally identified as a c-Cbl interacting protein by yeast two-hybrid screening [11]. Similarly, the family member CD2AP also binds to c-Cbl [37]. These interactions are regulated by the phosphorylation of c-Cbl. The majority of proteins that associate with CIN85/CD2AP regulate cell adhesion, migration and/or cancer cell invasion ([13], [38] and reviewed in [12]). Specifically, the CIN85 - c-Cbl complex has recently been shown to mono-ubiquitinate AMAP1 to drive invasion of breast cancer cells [19]. This complex is formed through the interaction of the SH3 domains of CIN85 with the proline-arginine motif PxxxPR of c-Cbl [11], [18]. The SH3 domains of CIN85 can also bind the PxxxPR motifs of ASAP1/AMAP1, Hip1R, SHIP1, and SH3KBP1-binding protein 1 (SHKBP1) [18], [39], [40]. The CIN85 PxpxxRh binding motif of LOX-PP represents a novel SH3 binding ligand. We showed that the binding of LOX-PP interfered with the CIN85 interaction with c-Cbl, and inhibited the invasive phenotype of MDA-MB-231 and NF639 breast cancer cells. These findings lead us to hypothesize that the interaction with LOX-PP compromises the functions of CIN85 that are essential for invasion by tumor cells. Interestingly, SH3KBP1 and the ORF3 protein of hepatitis E virus are also able to block the CIN85 - c-Cbl interaction leading to attenuated EGFR and Met receptor endocytosis, respectively, although the CIN85 domains of interaction were not mapped [41], [42]. The mass spectrometry analysis identified both family members – CD2AP and CIN85 – as binding partners for LOX-PP; however subsequent biochemical analysis revealed that the interaction with CIN85 is stronger and that CIN85 might bring CD2AP in to the complex (see Figure S2). Heteromerization could be mediated via the coiled-coil domain, as demonstrated previously [13]. While it has been speculated that CIN85 can modify the activity of CD2AP, the enhancement of the interaction of CD2AP with LOX-PP by co-expression of CIN85 is the first experimental evidence that this might in fact occur.

LOX-PP appears to interact exclusively with the SH3-B domain of CIN85. The superimposed Cα atoms of SH3-A and SH3-B from 53 paired residues have a relatively low r.m.s. deviation of 1.11 Ångstrom. Although the domains share only 47.1% sequence identity, the SH3 binding surfaces are very similar and align the key SH3 residues contacting the LOX-PP epitope, as discussed above. The main structural differences lie in the n-Src and RT loops flanking Arg116^LOX-PP^. The n-Src loop is the most divergent sequence region of the three CIN85 SH3 domains. Interestingly, in the SH3-B model there is a specific stabilizing hydrogen bond between Glu132^CIN85^ of the n-Src loop and the main-chain nitrogen of His117^LOX-PP^. In SH3-A this loop is positioned further from the peptide backbone in a distinct conformation and thus appears unable to form this connection. The spatial architecture surrounding Arg116^LOX-PP^ is modulated by these loops, and may provide a precise geometry for effective binding. Thus, deviations in the loops' positions found in SH3-A may prevent stable docking of the arginine. This is reminiscent of recent work showing how the structure of the n-Src loop of CIN85 SH3-A, but not of CD2AP SH3-A, is able to promote heterotrimer formation, that is, two SH3 domains clamping onto a single c-Cbl peptide, and thus giving rise to both class I and II ligand binding [43]. Other notable differences in coordinate positions include those of the C-terminal peptide region. Alternatively, the differential binding specificity may relate to local and/or longer-range electrostatic effects judging by the strikingly different charge distributions near to the binding groove of domains A and B as revealed in the computed electrostatic potential maps (see Fig. 5). Altered oligomerization states or solution-phase unfolding propensities [44] of these domains may also contribute, as well as the extent of stabilizing hydrogen bonds. Related issues have been explored experimentally in thorough biophysical studies with the SH3 domains from CD2AP [45] or CIN85 and known binding partners e.g. peptides derived from c-Cbl and CD2 [43], [45]. Importantly, it is possible that the LOX-PP epitope in the context of the full-length protein, not modeled here, may only be physically accommodated by the SH3-B domain due to these (electrostatic, structural, etc.) differences. Further experimental studies, including mutations and chimeric CIN85 SH3 domains are clearly required to fully understand this issue.

The lysyl oxidase enzyme is a key enzyme initiating collagen and elastin maturation via catalysis of the oxidative deamination of peptidyl lysine and hydroxylysine to peptidyl-α-aminoadipic-δ-semialdehyde in elastin and collagen chains. The consequent aldehydes lead to a spontaneous condensation forming inter- and intra-chain cross-links. This post-translational modification of extracellular matrix (ECM) molecules is critical for the collagen and elastin structural development. *Lox* knockout mice develop to term but die perinatally with poorly developed cardiovascular system and lungs [46], [47]. An association between organ fibrosis and increased LOX enzyme activity has also been documented [48]. Importantly, destruction of the appropriate collagen architecture promotes a more aggressive breast cancer phenotype [49], [50]. Here the interaction of LOX-PP with CIN85 is shown to inhibit the degradation of both collagen type I (gelatin) and type IV (Matrigel), suggesting a new role for the *LOX* gene in promoting the structural integrity of the ECM under normal conditions.

LOX-PP is very arginine-rich (13.5%), accounting for its cationic nature (pI = 12) [24]. It has been shown that arginine-rich proteins can enter cells via energy dependent- or independent endocytotic mechanisms and move to the endosomes from which they can leak into the cytoplasm [51]. Recently, we demonstrated that aa 26 to 100 region of intracellular LOX-PP interacts with Hsp70 and c-Raf to promote apoptosis and inhibit motility, potentially via altered mitochondrial function [28]. Previous structure prediction studies of LOX-PP using DISOPRED, GlobPlot and DisProt, and circular dichroism (CD) analysis have indicated that the propeptide assembles as an intrinsically disordered protein [5], [24], suggesting that LOX-PP does not have defined domains. Here, we identified the CIN85 binding motif at aa 111 to 119. Thus, multiple proteins, mediating various biological activities, are able to interact with various regions of LOX-PP and these associations appear to regulate the activities of these interacting proteins (e.g., affecting survival, motility, and invasive phenotype). In addition, a single-nucleotide polymorphism G473A (rs1800449) resulting in an Arg158Gln mutation has been shown to be associated with increased risk of estrogen receptor (ER)-alpha-negative invasive breast cancer in African-American women [6], and subsequently with increased risk of breast cancer and ovarian cancer in Chinese women [52], [53], and with gastric cancer [54]. It is reasonable to assume that the SNP regulates the function of LOX-PP and might affect its interaction with an associating protein(s). In summary, here LOX-PP is shown to interact directly with CIN85 via an atypical ligand thereby reducing interaction of CIN85 with c-Cbl, and reducing the invasive phenotype of breast cancer cells.

## Supporting Information

Figure S1 **Identification of CD2AP and CIN85 as LOX-PP interactors by co-purification/LC-MS/MS.** A summary of the analysis of the results of Mass Spectrometry of the 85-kDa band excised in Figure 1A is shown. Proteins co-purifying with LOX-PP were isolated as described in Figure 1A and the 85-kDa bands were analyzed by LC-MS/MS. Peptides from CIN85 (22) and CD2AP (42) were identified. Peptide sequences obtained from CIN 85 (top) and CD2AP (bottom) are highlighted in bold and underlined in the CD2AP and CIN85 amino-acid sequence. PPT.
(TIF)

Figure S2 **Ectopic CIN85 expression increases LOX-PP interaction with endogenous CD2AP.** GST (G) or LOX-PP-GST (L) protein was co-expressed with either GFP-CIN85 or GFP EV in HEK293T cells. GST-pull down assays were performed, as in Figure 1, and resulting extracts subjected to WB for ectopically expressed GFP and GST and for endogenous (Endo) CD2AP (Santa Cruz; B-4). Input, 4% of extracts. PPT.
(TIF)
