# Supplementary figures and images for: Inhibition of CIN85-Mediated Invasion by a Novel SH3 Domain Binding Motif in the Lysyl Oxidase Propeptide
Source: PLoS One. 2013 Oct 22;8(10):e77288. doi: 10.1371/journal.pone.0077288 (PMC3805583; doi:10.1371/journal.pone.0077288)

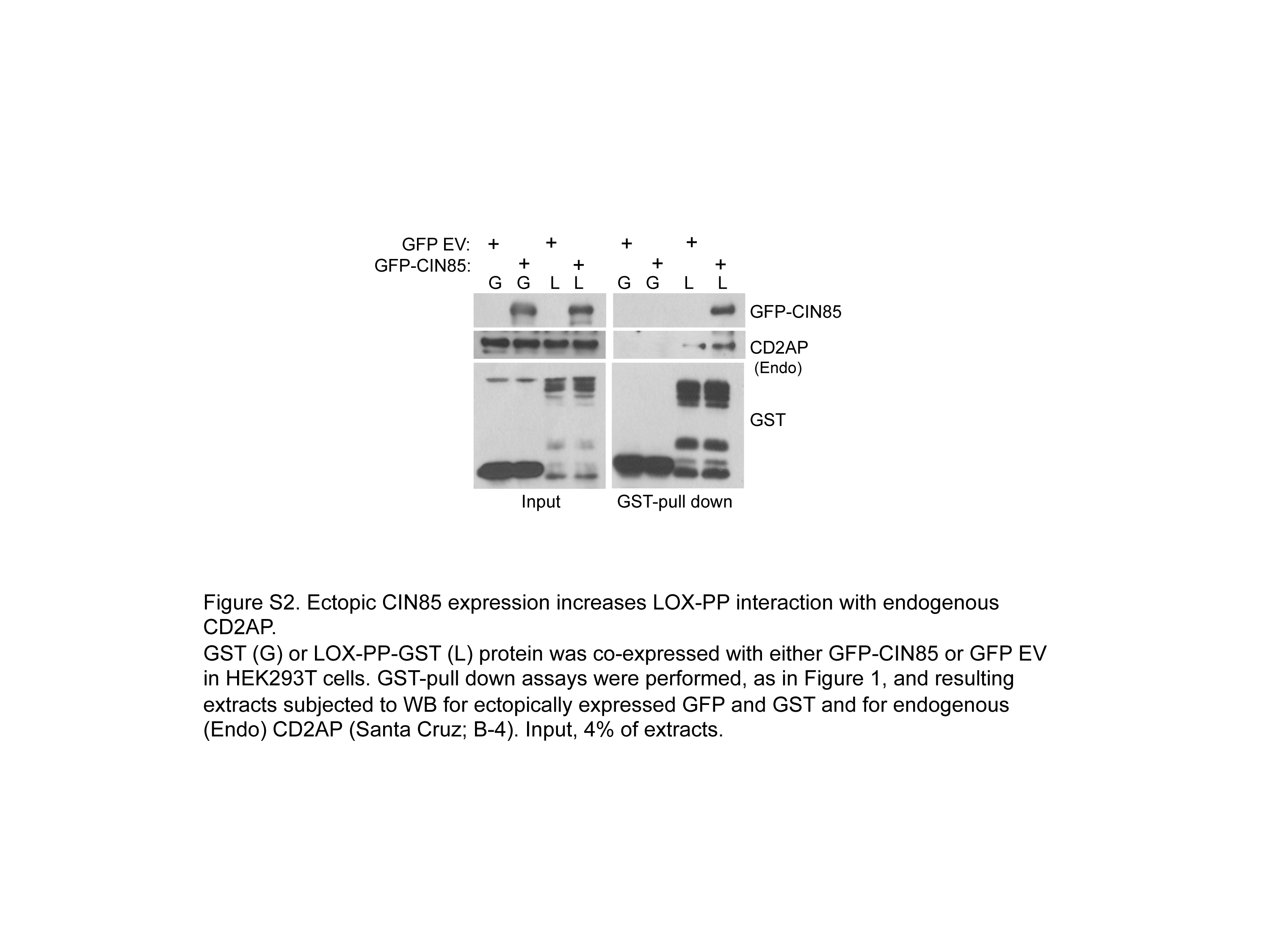

Supplement: Figure S2 — Ectopic CIN85 expression increases LOX-PP interaction with endogenous CD2AP. GST (G) or LOX-PP-GST (L) protein was co-expressed with either GFP-CIN85 or GFP EV in HEK293T cells. GST-pull down assays were performed, as in Figure 1, and resulting extracts subjected to WB for ectopically expressed GFP and GST and for endogenous (Endo) CD2AP (Santa Cruz; B-4). Input, 4% of extracts. PPT. (TIF) [file pone.0077288.s002.tif]
